# Complications des traitements traditionnels des traumatismes des membres au CHU Le Bon Samaritain de Walia (N’Djamena, Tchad)

**DOI:** 10.48327/mtsi.v2i1.2022.214

**Published:** 2022-02-15

**Authors:** Daniel Mossalbaye ADENDJINGUE, Madengar MOUASSEDE, Kodjalta MADJIREBAYE, Omar SALIA, Doudet Ossoga AMONÉ-NÉ

**Affiliations:** 1 CHU Référence nationale, BP 130 N’Djamena, Tchad; 2 CHU Le Bon Samaritain, BP 456 N’Djamena, Tchad; 3 CHU de Tengandogo, 11 BP 104 CMS Ouagadougou 01, Burkina Faso; 4 CNHU Hubert K. Maga, 01 BP 386 Cotonou, Bénin

**Keywords:** Traumatisme des membres, Fracture, Luxation, Complication, Traitement traditionnel, Hôpital, Tradipraticien, N’Djamena, Tchad, Afrique subsaharienne, Limb trauma, Fracture, Dislocation, Complication, Traditionnal treatment, Traditional healers, Hospital, N’Djamena, Chad, Sub-Saharan Africa

## Abstract

**Introduction:**

Le traitement traditionnel des traumatismes des membres par des tradipraticiens est omniprésent dans les sociétés africaines au sud du Sahara. Ces pratiques sont source de nombreuses complications. Cette étude se propose de préciser le profil de ces complications et d’identifier les facteurs favorisant le recours à ces tradipraticiens.

**Matériel et méthodes:**

Étude descriptive et analytique sur 12 mois, du 1er février 2018 au 31 janvier 2019, concernant tous les patients ayant consulté dans le service de chirurgie du Centre hospitalier universitaire Le Bon Samaritain de N’Djamena (CHUBS) pour une complication de fracture ou de luxation au niveau d’un membre qui avait été prise en charge par un tradipraticien. Le recueil des données a été fait à l’aide d’un questionnaire préétabli avec un recul moyen de 14 mois.

**Résultats:**

Sur 144 patients atteints de traumatismes des membres, 47 souffraient au moins d’une complication de fracture ou de luxation suite à un traitement traditionnel, soit 33 % des cas. Parmi eux, 32 ont été retenus pour l’étude. L’âge moyen était de 23 ± 3 ans (extrêmes de 10-61) et le sex-ratio de 2,6 en faveur des hommes. La provenance était majoritairement rurale. Les élèves/étudiants et les cultivateurs/éleveurs étaient les plus représentés. Les accidents de la circulation routière étaient les plus rencontrés (n = 20). L’influence de l’entourage (n = 14) a représenté le premier facteur conduisant au choix d’un traitement traditionnel. L’attelle en bois n’immobilisant pas les articulations proximales de la fracture, associée au bandage ischémiant à l’origine des gangrènes, a constitué le premier mode de contention (n = 15). Le délai moyen entre le traumatisme et le début du traitement traditionnel était de 8,5 heures. Le délai moyen entre le traitement traditionnel et la survenue des complications était de 106 jours (extrêmes de 1-302). L’œdème du membre, le cal vicieux, la gangrène et la pseudarthrose ont représenté les complications les plus fréquentes. La lésion initiale a été une fracture fermée dans la plupart des cas (n = 22) avec une prédominance des membres inférieurs (n = 22). La prise en charge hospitalière a été chirurgicale (n = 19) ou orthopédique (n = 13). L’évolution thérapeutique a été bonne, assez bonne ou mauvaise, respectivement dans 24, 2 et 6 cas.

**Conclusion:**

Les complications des traitements traditionnels des lésions traumatiques des membres sont graves. Une formation des tradipraticiens sur la connaissance des notions élémentaires d’immobilisation et la reconnaissance des signes de gravité, leur collaboration avec les structures de santé ainsi qu’une gratuité des soins dans les services hospitaliers permettraient de réduire ce phénomène. Les médias et réseaux sociaux devraient permettre de toucher un large public.

## Abstract (en)

**Introduction:**

Traditional treatment of limb trauma by traditional healers is ubiquitous in Sub-Saharan Africa. These practices are the source of many complications. This study aims to clarify the profile of these complications and to identify the factors favoring the consultation of traditional healers.

**Material and methods:**

Descriptive and analytical study over 12 months, from February 1, 2018 to January 31, 2019, covering all the patients who consulted at the surgery department of the CHU Le Bon Samaritain in N’Djamena (CHUBS) for a complication of a fracture or dislocation of a limb treated by a traditional healer. Data collection was carried out using a pre-established questionnaire with an average follow-up of 14 months.

**Results:**

Out of 144 patients, 47 (33%) suffered from at least one complication of fracture or dislocation following traditional treatment. Thirty-two were included in the survey. Their mean age was 23 years (range 10-61) and the sex ratio 2.6 in favor of men. The origin was mainly rural: pupils/students and farmers/breeders were the most represented. We identified individuals from all levels of education, mainly primary level. Road accidents were the most frequent (n = 20). The influence of the entourage (n = 14) was the first factor leading to the choice of traditional treatment. The wooden splint, which did not immobilize the proximal fracture joints, associated with the ischemic bandage causing the gangrene, was the first means of restraint (n = 15). The mean time between trauma and the start of traditional treatment was 8.5 hours. The mean time from traditional treatment to onset of complications was 106 days (range 1-302). The most common complications were swelling of the limbs, malunions, gangrene and pseudarthrosis. The initial lesion was a closed fracture in most cases (n = 22) with a predominance of the pelvic limbs (n = 22). Hospital management was surgical (n = 19) or orthopedic (n = 13). Therapeutic progress has been good, fairly good or bad regarding 24, 2 and 6 cases respectively.

The solicitation of traditional healers is frequent in traumatology. The influence of social background and ease of access to traditional “doctors” were noted as determining factors in the choice of this kind of treatment. The consequences of this practice have various origin: insufficient immobilization not respecting the standards; intense and untimely massages causing severe pain, secondary displacements and abnormal consolidation. Another study extended to patients who satisfied or not with the result of traditional fracture treatment in addition to those who consulted for complications would be more representative.

**Conclusion:**

The complications of traditional treatments for traumatic limb injuries are serious. The training of the traditional healers on basic notions of immobilization and the recognition of signs of seriousness, their collaboration with health structures as well as free care in hospital services would make possible to reduce this phenomenon. The media and social networks should help to reach a large audience.

## Introduction

La réduction des fractures est le domaine de thérapeutes spécialisés des médecines traditionnelles africaines [15], désignés en français par divers termes à connotation neutre, péjorative ou valorisante selon les interlocuteurs et les époques: « tradipraticiens », « rebouteux », « guérisseurs traditionnels »… Au Tchad, ils sont spécialisés et distincts des autres guérisseurs: ils sont appelés localement « tabibe attakhlidi » en arabe (littéralement « médecin traditionnel »), tandis que la pratique elle-même est désignée par « nam » en langue sara (littéralement « adaptation »). Le réducteur de la fracture procède en attachant des morceaux de bois ou des tiges comme attelles maintenues par des cordes (végétales, tissu ou plastique) autour de la zone traumatisée, tout en y appliquant des médicaments traditionnels, associés à des manipulations articulaires et des massages souvent vigoureux et très douloureux, parfois des scarifications [15]. Les savoir-faire de ces guérisseurs sont acquis le plus souvent par héritage familial, comme chez les Dogons du Mali par exemple [6]. La recherche par divination des causalités mystiques responsables de l’accident initial générateur du traumatisme des membres et leur neutralisation par la récitation d’incantations ou autres rituels n’est pas du ressort de tous.

Il n’y a pas de données officielles publiées par les autorités tchadiennes, mais une étude menée chez les enfants a noté que 71 % des amputations majeures ont été liées aux complications du traitement traditionnel des fractures [1].

Depuis les indépendances, la majorité des États africains ont mis en place des politiques de promotion des médecines traditionnelles avec l’aide des organisations internationales [10, 14]. Pourtant la médecine traditionnelle des fractures fait courir aux patients de grands risques de complications [19]. Les immobilisations sans respect des principes fondamentaux biomédicaux d’immobilisation des articulations voisines de la fracture favorisent les cals vicieux ou les pseudarthroses. Les massages intenses (étape préalable) sont souvent à l’origine d’importantes douleurs et de mobilisations de la fracture, source également d’anomalies de la consolidation. Les contentions trop serrées bloquant la circulation sanguine avec effet de garrot sont causes d’ischémie conduisant parfois à l’amputation. Les applications d’onguents de broyats végétaux humides ou les récitations de prières avec des crachats sur des lésions parfois ouvertes, les scarifications faites par des instruments non stériles sont à l’origine de complications infectieuses. La reprise prématurée de la marche est la cause des déplacements secondaires des fractures ou de l’absence de cicatrisation des lésions ligamentaires.

Dans cette étude, nous voulons faire ressortir le profil épidémiologique, clinique et thérapeutique de ces complications et identifier les raisons amenant à consulter les tradipraticiens.

## Matériel et Méthodes

Il s’agit d’une étude prospective descriptive et analytique concernant 32 patients, étalée sur une période de 12 mois allant du 1er février 2018 au 31 janvier 2019. La population d’étude a été constituée de tous les patients ayant consulté au Centre hospitalier universitaire Le Bon Samaritain (CHUBS) qui ont été suivis pour une complication dans les suites de traumatisme d’un membre pendant la période de l’étude, après avoir été traités traditionnellement.

Ont été inclus dans cette étude: les patients admis dans le service de chirurgie du CHUBS pour une complication de traumatisme d’un membre après avoir été traités initialement selon les pratiques traditionnelles, suivis jusqu’à la fin de l’étude et ayant accepté d’y participer.

N’ont pas été inclus: les patients présentant une complication de traumatisme d’un membre et n’ayant bénéficié d’aucun traitement traditionnel antérieurement, ceux ayant reçu initialement un traitement d’un agent de santé, et les patients perdus de vus. Les sujets ayant consulté pour d’autres pathologies ou dans les suites d’un traumatisme autre que celui d’un membre ont été exclus de l’étude.

Toutes les données ont été recueillies à l’aide d’un questionnaire préétabli. Le questionnaire a été rempli directement à partir des déclarations des patients ou de leurs accompagnants. Les numéros de téléphone des patients ou des accompagnants ont systématiquement été notés permettant de les joindre pour le suivi. Les variables étudiées ont été: l’âge, le sexe, la provenance, le niveau d’instruction, la profession, la nature de l’accident initial, la raison de choix du traitement traditionnel, le type de traitement traditionnel utilisé, le motif de consultation à l’hôpital, le délai entre le traumatisme et le début du traitement traditionnel, le délai d’installation des complications après le début du traitement traditionnel et d’admission à l’hôpital, l’état clinique à l’entrée, le type de lésion du membre traumatisé, les complications, le traitement hospitalier reçu, les résultats du traitement et le coût financier hospitalier.

Les critères d’appréciation des résultats sont regroupés selon des variables subjectives en: « bon » si guérison sans séquelles; « assez bon » si guérison avec séquelles fonctionnelles minimes permettant une fonctionnalité acceptable avec ou sans intervention chirurgicale; « mauvais » si amputation ou présence de séquelles fonctionnelles sévères ne permettant aucune amélioration fonctionnelle efficace même après intervention chirurgicale. Le recul moyen a été de 14 mois avec des extrêmes allant de 6 à 25 mois.

L’analyse des données a été faite grâce aux logiciels Microsoft Office Excel 2016 et SPSS version 18. Le test statistique réalisé a été celui de Chi2 de Pearson avec un seuil de significativité de 5 % (p < 0,05).

## Résultats

Durant la période d’étude, il y a eu 24 845 admissions au CHUBS, dont 1 074 en chirurgie: 144 pour traumatismes de membres, dont 47 souffrant au moins d’une complication de fracture ou de luxation suite au traitement traditionnel, soit 3 % des traumatismes des membres. Trente-deux personnes ont été retenues pour l’enquête et 15 n’ont pas été retenues (7 perdus de vue, 4 ont regagné leur village contre avis médical, 4 ont évoqué des raisons financières ou des difficultés d’hébergement en ville pour arrêter le suivi). L’âge moyen des patients était de 23 ± 3 ans (extrêmes de 10-61) et le sex-ratio de 2,6 en faveur des hommes (Tableaux I, II, III).

**Tableau I:** Répartition des patients en fonction de l’âge, du sexe et de la provenance Distribution of patients according to age, sex and origin

Âge		**n**
	0-15 ans	5
	16-30 ans	13
	31-45 ans	10
	plus de 45 ans	4
	**Total**	**32**
Sexe		**n**
	masculin	9
	féminin	23
	**Total**	**32**
Provenance		**n**
	rurale	
	urbaine	11
	**Total**	**32**

**Tableau II:** Répartition des patients en fonction du niveau d’étude, de la profession et du type d’accident Distribution of patients according to level of study, profession and type of accident

Études	non scolarisé	8
	primaire	9
	secondaire	8
	supérieur	7
Professions	élève/étudiant	13
	fonctionnaire	3
	cultivateur/éleveur	7
	commerçant	3
	autres*	6
Type d’accident	accident de la circulation	20
	accident domestique	4
	chute d’un arbre	4
	accident ludique	2
	rixe	2

* Autres: 2 conducteurs de taxi-moto; 2 femmes au foyer; 1 chauffeur et 1 retraité.

**Tableau III:** Répartition des patients en fonction de la raison de choix du traitement traditionnel et du type de traitement traditionnel Distribution of patients according to the reason for choosing traditional treatment and the type of traditional treatment

Raison de choix du traitement traditionnel	influence du milieu	14
	moyens financiers	5
	rapidité des soins	6
	popularité du tradipraticien	6
	absence de centre de soin	1
Type de traitement traditionnel	attelle + bandage	15
	attelle	11
	massage + bandage	4
	scarification + bandage	2

La consultation à l’hôpital a été motivée soit par la décision des proches (n = 15), soit par celle du patient lui-même (n = 14), ou encore après le conseil d’un personnel soignant, notamment l’infirmier de la zone (n = 3). Les patients ont consulté en urgence devant la douleur aiguë (n = 18), l’œdème (n = 7) et la suppuration locale (n = 1).

Le délai moyen entre le traumatisme et le début du traitement traditionnel était de 8,5 heures. Ce délai a été plus fréquemment de moins de 24 heures (n = 18) et dans la quasi-totalité des cas inférieur à 72 heures (n = 31).

La moyenne du délai entre le traitement traditionnel et la survenue des complications a été de 106 jours (extrêmes de 1-302). Dix-huit des patients ont consulté à l’hôpital 72 heures après l’installation des complications. Aucune personne n’a consulté dans les 24 heures.

L’état clinique des sujets à l’arrivée à l’hôpital était bon chez 30 personnes et altéré chez deux autres: un cas de suppuration locale et un de gangrène sèche vu à 23 jours avec une anémie normocytaire normochrome modérée et une perte pondérale non quantifiée.

La lésion initiale était une fracture fermée dans la plupart des cas (n = 22), une fracture ouverte (n = 6) et 4 luxations: une à l’épaule, deux au coude et une à la cheville avec fracture-luxation bimalléolaire fermée.

**Figure 1:**
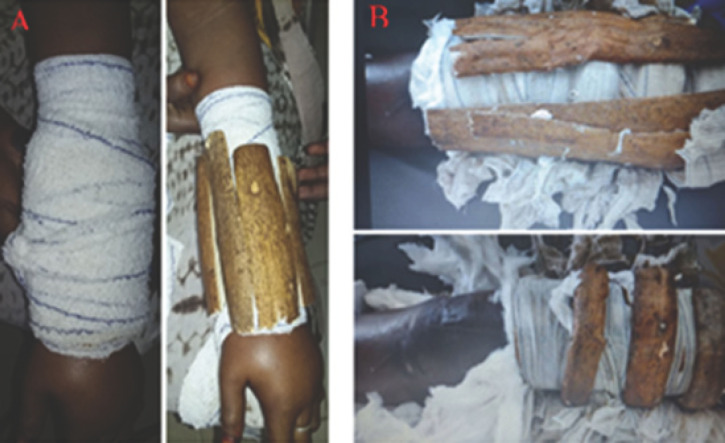
A. Important œdème de la main à J3 d’une immobilisation par attelle en bois avec bandage pour une fracture des deux os de l’avant-bras. Noter la présence de la bague. Une réduction a été réalisée, plus une ostéosynthèse par double plaque vissée après résorption de l’œdème. B. Même type d’immobilisation au niveau de la cuisse. A. Significant edema of the hand at D3 of immobilization by a wooden splint + bandage for a fracture of the two bones of the forearm. Note the presence of the ring. A reduction + osteosynthesis by 2 plates had been performed after resorption of the edema. B. Same type of immobilization in the thigh

Il a été retrouvé un œdème local et distal (Fig. 1) pour toutes les localisations de luxations. Le membre inférieur était le plus concerné (n = 22). Les types de complications observées (avec une prédominance de l’œdème) et le délai de leur apparition sont liés statistiquement (p = 0,04) (Tableau IV).

**Tableau IV:** Répartition des patients en fonction des complications et de leur délai d’apparition après le traitement traditionnel Distribution of patients according to complications and their average time after traditional treatment

	< 1 jour	1-2 jours	3-7 jours	8-45 jours	46-302 jours	Total
**Œdème**	5	3	6	-		14
**Suppuration locale**	-	-	-	1	-	1
**Gangrène sèche**	-		2	1		3
**Gangrène humide**	-	-	1	-		1
**Cal vicieux**	-	-	-		9	9
**Pseudarthrose**	-	-	-		4	4
**Total**	5	3	9	2	13	32

La première étape du traitement a consisté, dans tous les cas, à déposer l’immobilisation traditionnelle (Fig. 1 et 2), permettant un examen clinique local, puis une contention provisoire par une attelle plâtrée en fonction de la lésion et de son siège. La surélévation du membre était ensuite effectuée afin de permettre une détumescence en cas d’œdème et aussi dans un but antalgique. Un débridement a été d’emblée réalisé pour les lésions ouvertes. Le traitement définitif, qu’il soit orthopédique ou chirurgical, a été décidé en urgence ou en différé en fonction de la lésion. L’option thérapeutique chirurgicale a été retenue en cas de complications tardives, notamment de cal vicieux et de pseudarthrose (Fig. 3).

**Figure 2:**
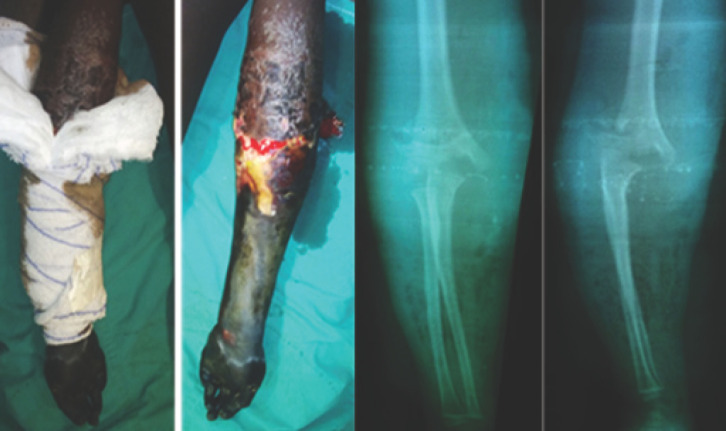
Gangrène mixte chez une enfant de 10 ans compliquant une fracture fermée de la palette humérale type IV de Rigault et Lagrange, traitée traditionnellement par attelle en bois. Une amputation trans-humérale a été réalisée Mixed gangrene in a 10-year-old child complicating a closed humeral paddle fracture type IV of Rigault and Lagrange traditionally treated with a wooden splint + bandage then bandage. A trans humeral amputation had been performed

**Figure 3:**
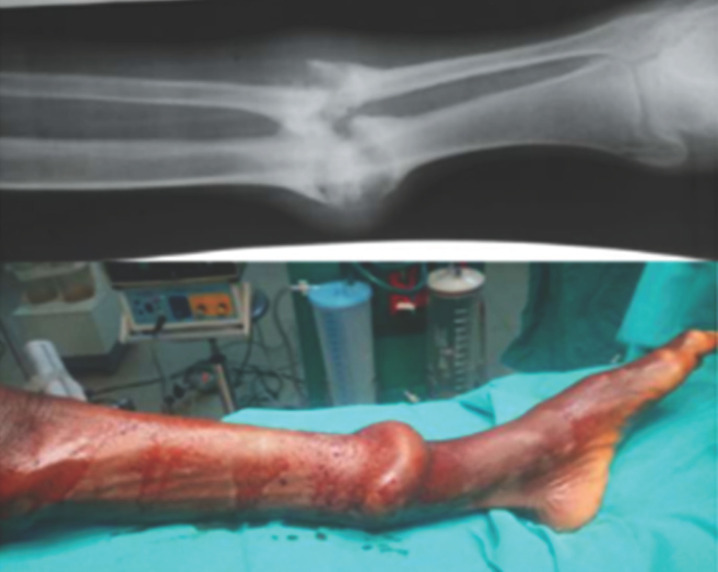
Pseudarthrose aseptique hypertrophique compliquant une fracture fermée des deux os de la jambe à 18 mois. Une cure de pseudarthrose et une ostéosynthèse par plaque vissée ont été réalisées Hypertrophic aseptic pseudarthrosis complicating a closed fracture of the two bones of the leg at 18 months treated by a cure for pseudarthrosis + osteosynthesis by plate

Ce traitement hospitalier a été orthopédique dans 13 cas, fait de 3 attelles plâtrées définitives et de 10 attelles plâtrées d’attente avant la pose d’un plâtre circulaire fenêtré ou non. Toutes les luxations ont été réduites à foyer fermé sous anesthésie générale à part la fracture-luxation bimalléolaire qui a bénéficié d’une réduction plus une ostéosynthèse. Le traitement chirurgical a été pratiqué chez 19 patients (Tableau V). Le résultat de suivi de nos patients en fonction de la présence ou non de séquelles est répertorié dans le Tableau VI. Aucun de nos patients amputés de membre n’a été appareillé. Aucun décès n’a été noté au cours de l’étude.

**Tableau V:** Répartition des patients en fonction du traitement chirurgical Distribution of patients according to surgical treatment

Fixateur externe	3
Plaque vissée	10
Clou centromédullaire	2
Amputation	4
**Total**	**19**

**Tableau VI:** Évolution thérapeutique en fonction de la présence ou non de séquelles et du type de traitement réalisé Therapeutic progress according to the presence or absence of sequelae and the type of treatment

	Bonne	Assez bonne	Mauvaise	Total
**Traitement chirurgical**	2	1	-	3
fixateur externe			
plaque vissée	10	-	-	10
clou centromédullaire	2	-	-	2
Amputation		-	4	4
**Total**	**14**	**1**	**4**	**19**
**Traitement orthopédique**	8	1	2	11
plâtre circulaire	2	-	-	2
attelle plâtrée	10	1	2	13
**Total**			

Le coût financier hospitalier de cette prise en charge a été forfaitaire et compris entre 120 et 360 euros avec une possibilité d’exonération partielle. Les autres types de dépenses (nourriture, arrêt de travail et déplacements du patient et de ses accompagnants, etc.) n’ont pas pu être évalués.

## Discussion

Les traumatismes des membres constituent un problème de santé publique et sont en augmentation à cause du développement du trafic routier [4]. Une étude béninoise a montré que 14 % des urgences chirurgicales sont des traumatismes des membres et qu’ils représentent 25 % des admissions en chirurgie [4].

La sollicitation des tradipraticiens est fréquente en traumatologie. Environ 80 % voire plus des populations rurales des pays en développement sont tributaires de cette médecine bien qu’elle soit pourvoyeuse parfois de séquelles importantes [5, 6, 20]. Ces séquelles sont liées à une immobilisation insuffisante d’un segment de membre siège de fracture ou de luxation ou à un appui prématuré sans tenir compte des caractéristiques anatomopathologiques de la lésion.

Cependant, le fait que les études menées dans ce domaine, y compris la nôtre, se focalisent sur les seules complications identifiées à l’hôpital, sans pouvoir déterminer la part des patients satisfaits ou non qui ne s’y sont pas présentés, constitue un biais d’appréciation pour préciser la part des complications dans les pratiques des tradipraticiens.

Cette série de 32 cas avec des complications a concerné essentiellement des sujets jeunes dont une importante part masculine. Ce constat est rapporté par d’autres auteurs d’Afrique subsaharienne [1, 7]. Cela s’expliquerait dans notre contexte par le fait que les hommes sont les plus grands usagers de la voie publique et les plus présents dans les tâches présentant davantage de risques physiques. Le niveau d’étude n’a pas semblé être un facteur déterminant, mais les couches sociales censées être les plus vulnérables (élèves/étudiants et paysans) et d’origine rurale sont les plus retrouvées. Au Bénin, Mensah et al [8] ont plutôt observé une prédominance de sujets de niveau primaire (47,7 %).

L’influence du milieu social et la facilité d’accès aux « médecins » traditionnels ont été notées comme déterminantes dans le choix de ce type de traitement. L’attirance des populations pour cette médecine est aussi consécutive à sa proximité spatiale, sociale et culturelle par rapport à la rareté des structures sanitaires, à leur accessibilité géographique et financière. Certaines études ont constaté comme raisons de choix du traitement traditionnel, l’influence de l’entourage familial, son coût financier abordable ou encore une information aux usagers jugée insuffisante de la part du personnel hospitalier [2,12,16,19]. Le fait que ces pratiques soient associées à une interprétation mystique, tant des circonstances du traumatisme initial que des événements biologiques et non biologiques concomitants affectant le blessé et ses proches, constitue aussi un mobile d’attirance pour les usagers.

**Figure 4:**
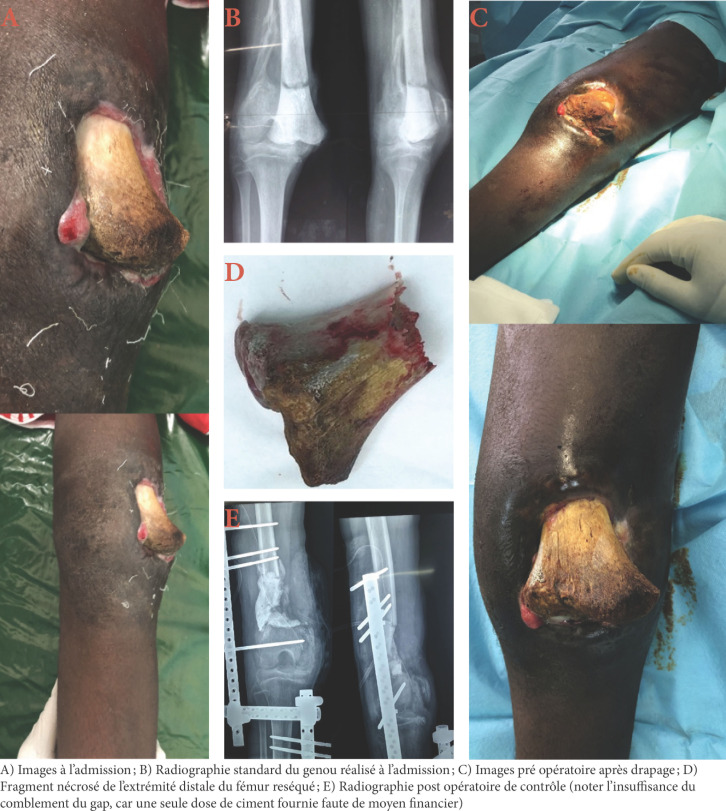
Patient de 15 ans, de provenance rurale, reçu à 3 mois d’une fracture-décollement épiphysaire type I de Salter et Harris ouverte du fémur distal droit, traitée traditionnellement avec exposition osseuse en médial, compliquée d’une surinfection avec nécrose osseuse. Il a été réalisé une résection osseuse, un prélèvement pour l’examen cytobactériologique, un débridement, une exofixation fémoro-tibiale, un comblement par spacer (ciment) réalisant la technique de la membrane induite et un drainage. 15-year-old patient of rural origin, received at 3 months with an open Salter and Harris type I epiphyseal fracture-detachment of the right distal femur, traditionally treated with bone exposure medially, complicated by superinfection with bone necrosis. Bone resection, a sample for cytobacteriological examination, debridement, femoro-tibial exofixation, spacer (cement) filling using the induced membrane technique and drainage were performed.

Nos patients n’ont consulté à l’hôpital qu’en cas de complications devenues intenables et le plus souvent lorsque sont apparues une douleur importante et une inquiétante tuméfaction du membre (Fig. 1). Les tuméfactions douloureuses et les gangrènes ischémiques (Fig. 2) retrouvées sont liées à une immobilisation par des baguettes de bois (de tous genres dans notre cas) disposées circulairement le long du membre. Ces baguettes de bois sont censées ponter le foyer présumé de la fracture sans immobiliser les articulations voisines et sont maintenues par un bandage souvent trop serré (Fig. 1). Les massages intenses et intempestifs sont souvent à l’origine d’importantes douleurs et de mobilisations de la fracture, source également d’anomalies de la consolidation (Fig. 3). Notre seul cas enregistré de gangrène humide a fait suite à une fracture ouverte. Il faut noter que tous les cas de gangrène ont conduit à une amputation du membre concerné. Ces amputations sont pratiquées au bras, à la cuisse et à la jambe. Tékpa et al [18] ont recensé au Sénégal, sur une période inférieure à la nôtre, 21 cas de gangrène chez des enfants alors qu’une étude faite en Gambie en a noté 9 en 29 mois [19]. Pour les fractures ouvertes, l’absence d’antibioprophylaxie, de l’impératif parage ou encore de la couverture osseuse de la part des tradipraticiens conduit souvent à des complications nécessitant le recours à la chirurgie réparatrice (Fig. 4). Le choix de la chirurgie a prédominé dans notre cas pour le membre inférieur, ce qui pourrait s’expliquer par le handicap de la locomotion souvent plus mal supporté par les patients qu’une lésion du membre supérieur.

Au terme de notre étude, le résultat thérapeutique a été bon chez la majeure partie des patients avec une réinsertion socio-professionnelle acceptable. Une étude faite au Niger concernant le traitement chirurgical chez 61 patients a noté un résultat légèrement supérieur au nôtre avec 86,88 % de bons résultats tout en incluant les 4 perdus de vue [17].

La disponibilité et l’accès aux structures sanitaires restent des points cruciaux pour la

A) Images à l’admission; B) Radiographie standard du genou réalisé à l’admission; C) Images pré opératoire après drapage; D) Fragment nécrosé de l’extrémité distale du fémur reséqué; E) Radiographie post opératoire de contrôle (noter l’insuffisance du comblement du gap, car une seule dose de ciment fournie faute de moyen financier) résolution de ce problème de santé publique. L’efficacité des mesures ne passe pas par les seules stigmatisations et incriminations de ces pratiques traditionnelles, mais par la sensibilisation des acteurs et des usagers aux risques encourus [8,12,13,19]. Beaucoup de pays africains, dont le Tchad, reconnaissent officiellement la médecine traditionnelle. Celle-ci est encouragée et soutenue par l’État tchadien amenant les tradipraticiens à se regrouper en associations. Une division de la pharmacopée et de la médecine traditionnelles, directement rattachée au ministère en charge de la santé publique, a été créée afin de promouvoir cette médecine. Or, l’absence de textes règlementant la profession ne permet pas d’identifier des critères officiels de reconnaissance de ses membres, ce qui permettrait un meilleur encadrement de leur pratique.

## Conclusion

Les complications découlant du traitement traditionnel des lésions traumatiques sont graves avec parfois un risque vital. Les conséquences socio-économiques considérables imposent une sensibilisation large incluant tous les acteurs, notamment les tradipraticiens, la population, les autorités administratives ainsi que le personnel soignant. Cela passerait par la promotion des associations de tradipraticiens (tout en tenant compte de la difficulté liée à l’ancrage de cette pratique au sein de la société) et l’utilisation des médias et réseaux sociaux pour atteindre un large public. Nous plaidons pour un encadrement de cette pratique traditionnelle par des textes règlementaires et une sensibilisation de ses acteurs axée sur les notions élémentaires de l’immobilisation, de la reconnaissance des signes de gravité, et de la nécessité de collaborer avec les structures de santé aboutissant à la reconnaissance de leur compétence. En outre, une couverture sanitaire généralisée à toute la population ou une prise en charge gratuite des patients reste une pièce fondamentale pour réduire ce phénomène.

## Liens d’intérêts

Les auteurs ne déclarent aucun lien d’intérêt.

## Contribution des auteurs

D. M. ADENDJINGUE: analyse des données, interprétation des données, rédaction du manuscrit, finalisation

M. MOUASSEDE: conception de l’étude, supervision de l’étude, relecture et validation du manuscrit

K. MADJIREBAYE: prospection bibliographique, collecte des données, saisie des données

O. SALIA: lecture et correction

D. O. AMONÉ-NÉ: correction
